# The Introgression of RNAi Silencing of γ-Gliadins into Commercial Lines of Bread Wheat Changes the Mixing and Technological Properties of the Dough

**DOI:** 10.1371/journal.pone.0045937

**Published:** 2012-09-24

**Authors:** Javier Gil-Humanes, Fernando Pistón, María J. Giménez, Antonio Martín, Francisco Barro

**Affiliations:** Department of Plant Breeding, Institute for Sustainable Agriculture (CSIC), Córdoba, Spain; TGen, United States

## Abstract

In the present work the effects on dough quality by the down-regulation of γ-gliadins in different genetic backgrounds of bread wheat were investigated. RNAi-mediated silencing of γ-gliadins was introgressed by conventional crossing into three commercial bread wheat lines (namely ‘Gazul’, ‘Podenco’ and ‘Arpain’), and along with the transgenic line A1152 (cv. Bobwhite) compared with their respective wild types. The protein fractions were quantified by RP-HPLC, whereas the technological and mixing properties were assessed by SDSS test and by the Mixograph instrument. Principal component analysis (PCA) was carried out for both the wild types and the transgenic lines, showing differences in the factors affecting the technological and mixing properties of the dough as a consequence of the reduction of the γ-gliadins. In transgenic lines, the α- and ω-gliadins, and total gliadins negatively affected the dough strength and tolerance to over-mixing, whereas the L/H ratio showed the opposite effect, positively influencing the dough quality. The increase of the SDSS volume in the transgenic lines of ‘Gazul’, ‘Podenco’ and ‘Arpain’ indicates increased gluten strength and quality respect to the wild types. SDSS volume was found to be positively influenced by the amount of glutenins, which were also increased in the transgenic lines. In addition, a positive effect was observed in the MT, PR1 and RBD in some of the transgenic lines of ‘Podenco’ and ‘Arpain’. In conclusion, the down-regulation of γ-gliadins resulted in stronger doughs and a better tolerance to over-mixing in some transgenic lines. Although the reduction of γ-gliadins seems not to have a direct effect on the mixing and bread-making properties, the compensatory effect on the synthesis of the other prolamins may result in stronger doughs with improved over-mixing resistance.

## Introduction

Bread-making quality of wheat is strongly influenced by gluten proteins (also called prolamins), which are typically classified into glutenins and gliadins. Glutenins consist of two different groups of subunits based on their separation in a sodium dodecyl sulphate polyacrylamide gel electrophoresis (SDS-PAGE) system, the high molecular weight (HMW) and the low molecular weight (LMW) subunits. The HMW glutenins form complex polymers stabilised by inter-chain disulphide bonds and are mainly related with dough elasticity. Bread wheat contains six HMW glutenin subunits (HMW-GS) genes, with tightly linked pairs of genes encoding x- and y-type subunits that are present at each of the *Glu-A1*, *Glu-B1*, and *Glu-D1* loci on the long arms of chromosomes 1A, 1B, and 1D, respectively. However, varieties of wheat only express between three and five HMW-GS. Gliadins are classed into three structural types: α-, γ- and ω-gliadins according to their mobility in acidic electrophoretic systems (A-PAGE), and have specific water-retaining capacities important for dough viscosity. In contrast to glutenins, gliadins are monomeric components that contribute mainly to the extensibility and viscosity of the dough [1]. Wheat gliadin genes occur in tightly-linked clusters, termed blocks, located at complex loci on group 1 and 6 chromosomes. The ω- and γ-gliadins are coded by clusters of genes at the *Gli-1* loci (*Gli-A1, Gli-B1, Gli-D1*) on the short arms of the homologous group 1 chromosomes and are tightly linked to the LMW-GS genes on the *Glu3* loci [2], whereas the α-gliadins are controlled by the *Gli-2* loci (*Gli-A2*, *Gli-B2*, *Gli-D2*) present on the short arms of the group 6 chromosomes [3].

The HMW glutenins are considered major determinants of bread quality as allelic differences in the HMW-GS composition influence the structure and properties of the glutenin polymers [3], [4]. Consequently, the amount and relative proportion of HMW-GS are important factors in functional performance for bread-making quality [5]. Although gliadins are related to loaf volume [6], [7], the contribution of individual gliadins to the dough quality is not well understood [8] as they are encoded by large multigene families and inherited in blocks. In addition, LMW glutenin subunit genes are linked to some gliadin genes, so that the effects of gliadins on quality are difficult to interpret [9].

Glutenins and gliadins have been recurrent targets in genetic transformation of wheat to study the properties of individual proteins or groups of proteins and their contribution to the mixing and baking properties of the dough. Different HMW-GS correlated with a good quality of the dough (namely 1A×1, 1D×5 and 1Dy10) have been expressed or over-expressed in transgenic wheat resulting in increased strength and elasticity [10]–[14]. The LMW-GS have been also over-expressed in bread wheat with contradictory results, either promoting a decrease of the SDS sedimentation value [15] or resulting in stronger and more stable doughs in some of the transgenic lines [16]. Yue et al. [17] also obtained transgenic wheat lines with down-regulation of the 1D×5 subunit by RNAi, leading to a decrease of the flour quality based on Farinograph, gluten and Zeleny tests. On the other hand, gliadins have constituted a target for RNAi-mediated gene silencing due to their involvement in the development of the celiac disease and the interest in developing wheat lines with reduced contents of gliadins suitable for the celiac community. The contents of α-gliadins [18], γ-gliadins [19] and all α-, γ- and ω-gliadins [20] have been reported to be strongly down-regulated in bread wheat by RNAi. The silencing of α-gliadins in bread wheat resulted in increased dough strength and slightly decreased loaf volume [21], whereas the silencing of the γ-gliadins did not produce significant changes in the mixing and pasting properties of the bread wheat cv. Bobwhite as determined by the Mixolab [22] and by the Mixograph [23], indicating that γ-gliadins may not have a major role in dough mixing properties of wheat flour.

In the present work the γ-gliadins silencing was introgressed by conventional crossing into three commercial bread wheat lines (namely ‘Gazul’, ‘Podenco’ and ‘Arpain’). The effects of the γ-gliadin silencing on protein composition and mixing properties in the different genetic backgrounds were investigated. Interactions between the rearrangements in the protein composition and their influence on the dough quality are discussed independently for the wild-types and transgenic lines.

## Materials and Methods

### Plant Material

A set of seven transgenic lines with reduced levels of γ-gliadins by RNA interference, and four wild-type lines were used in the present work. Transgenic line A1152 was obtained by genetic transformation of *T. aestivum* cv. Bobwhite (‘BW208’) as reported Gil-Humanes et al. [19] and described in Pistón et al. [23]. Transgenic lines G613, G622, G626, G845, G658 and G664 were obtained by crossing line A1152 and each of the commercial bread wheat lines ‘Gazul’, ‘Podenco’ and ‘Arpain’ as follows: G613 and G622 were the result of the A1152×‘Gazul’ crossing; G626 and G845 were obtained from the crossing A1152×‘Podenco’; and G658 and G664 from the crossing A1152×‘Arpain’. The F1 population from each crossing was backcrossed once with the respective commercial line, and then self-pollinated for three generations. Homozygous progeny of plants containing the γ-gliadins down-regulation as well as the HMW-GS and LMW-GS profiles corresponding to the parental commercial lines were identified by A-PAGE and SDS-PAGE of gliadins and glutenins, respectively, by single half-seed descendent.

### Grain Traits

Thousand seeds weight (g) was determined to 1000 seeds from each sample. Test weight (g l^−1^) was calculated by weighing 100 ml of cleaned grains from each sample. Two technical measurements were carried out for each sample.

### Total Protein and Starch

The protein content of whole flour was calculated from the Kjeldahl nitrogen content (%N×5.7) according to the standard ICC method no. 105/2 [24], and the starch content was determined according to the standard ICC method no. 123/1 [25]. Both parameters were expressed on a 14% moisture basis.

### Sodium Dodecyl Sulphate Sedimentation (SDSS) Assay

The SDS sedimentation volume was determined as described by Williams et al. [26]. Three technical replicates were carried out for each biological sample.

### Reversed-phase High-performance Liquid Chromatography (RP-HPLC)

The gliadin fraction was extracted from 100 mg of flour stepwise three times with 670 µl of 60% (v/v) ethanol, vortexing for 2 min and incubation at RT 10 min with shaking. Samples were centrifuged at 6,000×*g.* for 20 min, supernatants were collected and mixed all together. The glutenin fraction was extracted from the insoluble material of the previous step stepwise two times with 500 µl of 50% (v/v) 1-propanol, 2 M urea, 0.05 M Tris-HCl (pH 7.5) and 2% (w/v) DTT, vortexing for 2 min at RT and incubation for 15 min at 60°C with shaking. Samples were centrifuged at 6,000×*g.* for 20 min; supernatants were collected and mixed all together. Finally, samples were filtered through a 0.45 µm nylon filter (Teknokroma, Barcelona, Spain). Gliadin (40 µl) and glutenin (40 µl) extracts were applied to a 300SB-C8 reverse phase analytical column (4.6×250 mm, 5 µm particle size, 300 Å pore size; Agilent Technologies, Santa Clara, CA) using a 1200 Series Quaternary LC System liquid chromatograph (Agilent Technologies, Santa Clara, CA) with a DAD UV-V detector, as described Wieser et al. [27]. Absorbance was monitored with the DAD UV-V module at 210 nm. The integration procedure was handled automatically by the software with some minor manual adjustment. Absolute amounts of gliadin and glutenin fractions were determined using bovine serum albumin (BSA; BSA ≥98%, fraction V., Sigma-Aldrich, St. Louis, MO) as standard. Three independent repetitions were carried out for each transgenic line and wild type.

### Polyacrylamide Gel Electrophoresis

Two mature seeds were crushed into a fine powder and used to extract the endosperm storage proteins. Gliadins were extracted from the flour with a 5∶1 (µl mg^−1^ flour) ratio solution of 1.5 M N,N-Dimethylformamide, 20% (w/v) sucrose for 45 min at room temperature (RT) with shaking. Samples were centrifuged for 20 min at 13,000×*g.* and supernatants loaded and separated in A-PAGE gels for 1 h 15 min at 15°C and 40 mA per gel. Gel composition was as described in Khan et al. [28] with some minor modifications: 3.8×10^−4^ ferrous sulphate instead 2.1×10^−4^ and 0.125% (w/v) aluminium lactate instead of sodium lactate. Pellets from the previous step were washed two times with a 50% (v/v) aqueous 1-propanol solution during 30 min at 60°C, and centrifuged for 10 min at 13,000×*g.* Supernatants were discarded and glutenins extracted with a 5∶1 (µl mg^−1^flour) ratio solution of 0.08 M TRIS-HCl, pH 8.5, 50% (v/v) 1-propanol, 2% (w/v) dithiothreitol (DTT) during 30 min at 60°C with vortexing. Samples were centrifuged for 10 min at 13,000×*g.* and cold acetone was added to the supernatants in a ratio of 5∶1 (v/v) and incubated overnight at −20°C. Samples were centrifuged for 10 min at 13,000×*g.* and the precipitated glutenins resuspended in a 5∶1 (µl mg^−1^ flour) ratio solution of 0.125 M TRIS-HCl, pH 6.8, 2% (w/v) SDS, 2% (w/v) DTT, 10% (v/v) glycerol, 0.002% (w/v) Bromophenol Blue and incubated for 30 min at 60°C with continuous vortexing. Glutenin extracts were loaded and separated in SDS-PAGE gels for 5 h and 30 min at 10°C and 15 mA per gel. Tricine electrophoresis buffer and acrylamide stacking and resolving (T = 10%, C = 1.28) gels were as described by Shewry et al. [29]. A-PAGE and SDS-PAGE gels were stained with a solution of 0.05% (w/v) Coomassie Blue R-250, 5% (v/v) ethanol, 12% (w/v) trichloroacetic acid and then immersed in tap water overnight to remove excess of stain.

### Mixograph Analysis

Dough mixing properties were determined with a 35 g Mixograph (National Manufacturing Co., Lincoln, NE). Prior to milling, kernel moisture was adjusted to 14% overnight at room temperature with continuous shaking. Milling was carried out in a Cyclotec™ 1093 mill (Foss Analytical, Hillerød, Denmark), and then flour was refined through a 250 µm screen. Optimum water absorption of each sample was determined following the AACC 54-40A method [30], adapting the proposed equation by using a proportional mixture of the different samples of flours used in the present work. The mixing parameters determined were mixing time (MT), peak resistance (PR1) or height of the centre curve at the peak point, peak width (PW1) or width of the curve at the peak point, height of the curve at three minutes after the peak (PR3), width of the curve at three minutes after the peak (PW3), and resistance breakdown (RBD) or percentage of reduction of the height between the peak point and three minutes after the peak. Three independent repetitions were carried out for each transgenic line and wild type, corresponding to each block of the assay.

### Statistical Design

The lines described in the present work were assayed using a randomised complete block design with three replicates of five plants each. Data were analysed with the statistical software R version 2.12.1 [31] using the Graphical User Interface (GUI) R Commander. The randomised design was generated with the package *agricolae* (function *design.rcbd*). Major assumptions of analysis of variance (ANOVA) were confirmed by the Shapiro-Wilk’s test for normal distribution (function *shapiro.test*, package *stats*) and by the Levene’s test for homogeneity of variances (function *leveneTest*, package *car*); and variables were transformed if required. The differences in the data were assessed using analysis of the variance (ANOVA) (function *aov*, package *agricolae*), followed by LSD *post hoc* all-pairwise comparisons test (function *LSD.test*, package *agricolae*). The statistical analysis between the different lines was carried out by using the ANOVA model “Variable ∼ Line + Block”, whereas to compare the transgenic and the control (wild-type) lines we used the model “Variable ∼ Type + Block”, where the factor ‘Type’ had 2 levels: transgenic and control. In all the statistical analyses *P* values lower than 0.05 were considered significant, and lower than 0.01 were considered highly significant. Principal component analysis (PCA) was carried out for multivariate statistical analysis of protein composition and quality parameters (SDSS and Mixograph values) with the SPSS version 11.0 statistical software package (SPSS Inc., Somers, NY). No rotation was applied to extract the principal components. The correlation matrix was also obtained for the studied data, and the correlations with a *P* value lower than 0.1 were considered significant.

## Results

### A-PAGE and SDS-PAGE Analysis

**Figure 1:**
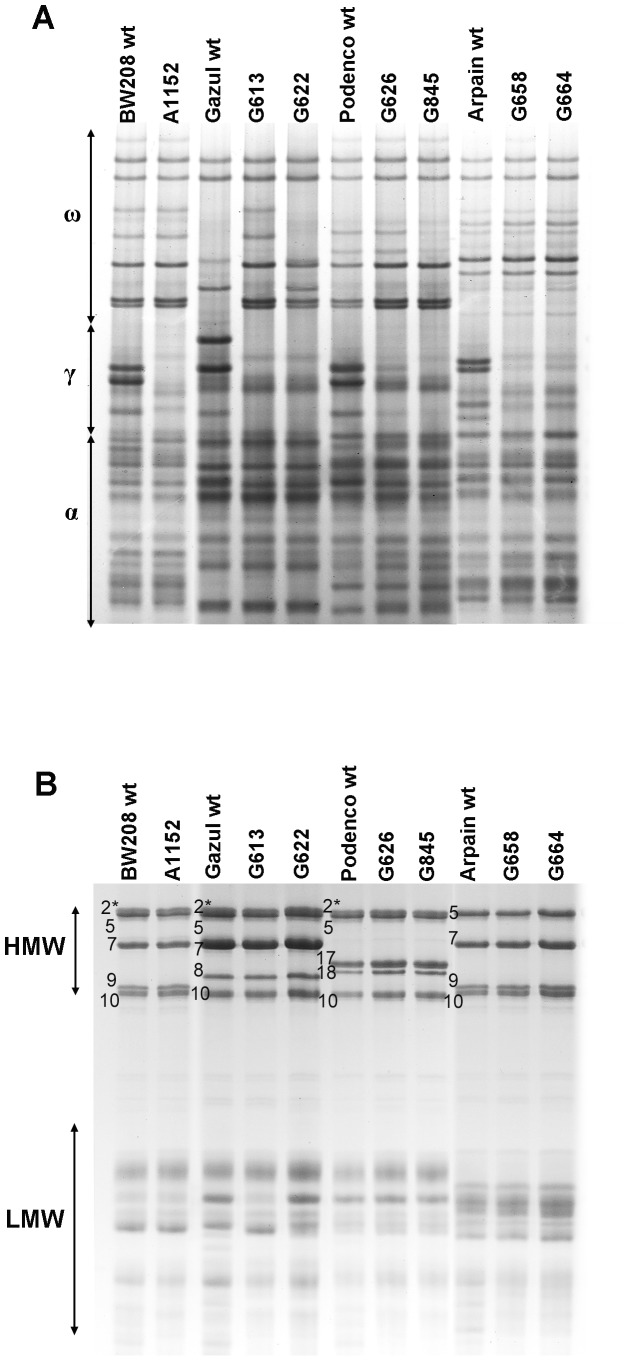
A-PAGE and SDS-PAGE gels. A-PAGE of gliadins (A) and SDS-PAGE of glutenins (B) of the parental lines used (A1152 and the wild types ‘Gazul’, ‘Podenco’ and ‘Arpain’), the resulting hybrid transgenic lines and the wild-type cv. Bobwhite (‘BW208’). HMW subunits are labelled on gels of panel B.

As observed in Figure 1, all the four wild types have a different gliadin profile (Fig. 1A), with only ‘Podenco’ showing a similar γ-gliadin and ω-gliadin profiles to the wild type ‘BW208’. RNAi is a dominant character, and as a result, all the lines of the F1 population obtained from the crossing of the line A1152 and the three commercial lines presented down-regulation of the γ-gliadins (data not shown). Following, the F1 hybrids were backcrossed with the corresponding parental commercial lines and then self-pollinated, selecting in each generation both the silencing of γ-gliadins and the glutenins profile of the parental line. After three generations of self-pollination, the gliadin and glutenin profiles of the resulting lines were as shown in Figure 1A and 1B, respectively. All transgenic lines presented the same profile of α- and ω-gliadins than the corresponding parental line, except lines G613 (obtained from the crossing×‘Gazul’), whose ω-gliadins were as the cv. Bobwhite, and line G622, with a mixture of ω-gliadins belonging to genotype ‘Gazul’ and cv. Bobwhite (Fig. 1A). Figure 1B shows that the wild type ‘BW208’ (cv. Bobwhite) contains the HMW-GS A×2*, B×7+By9 and D×5+Dy10, whereas ‘Gazul’ has the combination A×2*, B×7+By8 and D×5+Dy10, ‘Podenco’ the A×2*, B×17+By18 and D×5+Dy10 and ‘Arpain’ the B×7+By9 and D×5+Dy10. The HMW-GS profiles of the six transgenic lines (G613, G622, G626, G845, G658 and G664) contained the same subunits than the respective commercial lines described above (Fig. 1B). Line G613 presented LMW-GS bands corresponding to the cv. Bobwhite instead of ‘Gazul’. All the lines were homozygous for the gliadins and glutenins as demonstrated the A-PAGE and SDS-PAGE analysis carried out to 20 seeds of each generation (data not shown).

### Grain Composition Analysis

Table 1 shows the characterization of the protein and starch composition of the wild types (‘BW208’, ‘Gazul’, ‘Podenco’ and ‘Arpain’). The analysis of the variance (ANOVA) showed significant differences in all the fractions of gliadins and glutenins, and also in the total prolamin and protein content, but not in the total starch content. The LSD analysis of homogeneous groups (*p*<0.05) did not show significant differences for the content of ω- and α- gliadins, whereas for the γ-gliadins content and for the total content of gliadins a higher variability was found. The glutenin composition (LMW and HMW) also showed significant differences between the wild types, with higher amounts of total glutenins in the lines ‘Gazul’ and ‘Arpain’. The total content of prolamins (gliadins plus glutenins) showed only differences between lines ‘Gazul’ and ‘Podenco’, whereas the total content of proteins was only different in ‘Gazul’. The protein and starch composition was also determined to the transgenic lines (Table S1). A representative RP-HPLC chromatogram of gliadins and glutenins extracts from transgenic and wild-type lines is represented in Figure S1.

**Table 1:** Protein and starch composition of the wild-type lines.

	Gliadin (µg mg^−1^ flour)				Glutenin (µg mg^−1^ flour)			Prolamins(µg mg^−1^ flour)	Total protein (%)	Total starch (%)
Line	ω	α	γ	Total	LMW	HMW	Total			
**BW208**	6.79 ab	26.64 ab	21.00 b	54.42 ab	8.78 b	4.38 d	13.16 b	67.58 abc	12.23 b	56.41
**Gazul**	7.03 ab	29.51 ab	28.43 a	64.97 a	14.96 a	8.41 ab	23.37 a	88.34 a	13.81 a	52.73
**Podenco**	4.8 b	18.73 b	17.52 b	41.06 bc	8.69 b	4.54 cd	13.24 b	54.29 c	11.69 b	57.90
**Arpain**	6.13 ab	24.3 ab	16.27 b	46.71 abc	15.23 a	6.69 bcd	21.92 a	68.62 abc	11.67 b	56.76

Means within a column followed by the same letter are not significantly different at *p*<0.05, as determined by the LSD all-pairwise comparisons test.

**Figure 2:**
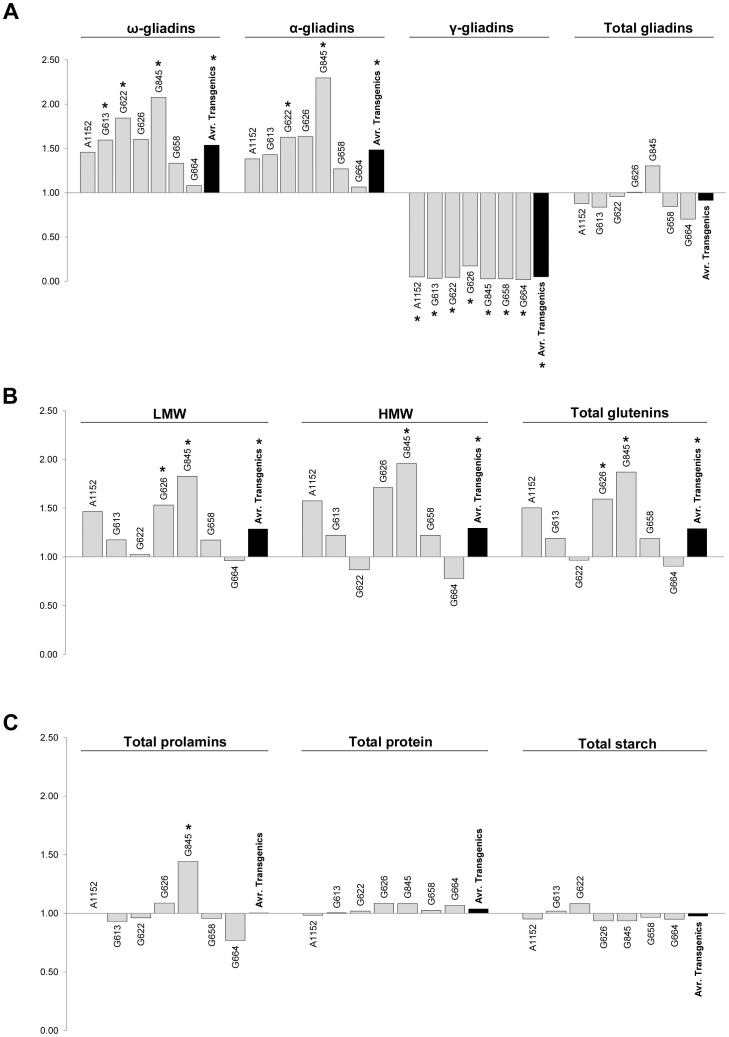
Ratio transgenic/wild type for the seed composition parameters of each transgenic line. The ratio transgenic/wild type (obtained by dividing the value of the transgenic line and the value of its corresponding wild-type line) is represented for each parameter described in Table 1. Values above 1 represent higher values of the transgenic than the control, whereas values below 1 represent the opposite. Asterisks over a bar indicate that the transgenic line presents a significantly different value (*p*<0.05) respect to its corresponding wild type as determined by the LSD all-pairwise comparisons test.

As showed in Figure 2A, the average of transgenic lines presented significantly higher contents of ω- and α-gliadins, whereas the fraction of γ-gliadins was significantly lower in all the transgenic lines respect to the wild-types. As result, total gliadins were not significantly affected in any of the transgenic lines (Fig. 2A).

Similarly to the gliadin fractions, the glutenins behaviour was very similar among the transgenic lines, with most of them presenting higher contents of LMW, HMW and total glutenins (Fig. 2B). However, the statistical analysis showed that only lines G626, G845 and the average of the transgenic lines had significantly higher contents of LMW and total glutenins than the respective wild types, whereas for the HMW, only the line G845 and the average of transgenic lines were significantly higher (Fig. 2B).

Total prolamins, total proteins and total starch were not statistically different between transgenic lines and their respective wild types; and only the line G845, presented a significantly higher value for the total prolamins content with respect to its corresponding wild-type ‘Podenco’ (Fig. 2C).

### Analysis of the Dough Quality

Dough quality was studied in all the lines, and the differences were assessed using ANOVA followed by LSD *post hoc* all-pairwise comparisons test (Table 2). Results showed that only three lines presented significantly different values for the weight of 1000 seeds and the test weight. No significant differences were found for the L/H ratio in any of the transgenic lines, whereas the Gli/Glu ratio was found to be significantly lower in line A1152 and in the average of transgenic lines (Table 2). On the other hand, the SDSS volume showed an increase in all the transgenic lines with respect to the wild types, resulting in significantly higher volumes for transgenic lines G613, G626, G845 and G664, and for the average of transgenics. Figure S2 shows the seeds of each transgenic and wild-type line.

**Table 2:** Quality parameters of transgenic and wild-type lines.

Line	1000 seeds (g)	Grain test weight (g/L)	L/H	Gli/Glu	SDSS	Mixograph					
					(ml)	MT (s)	PR1 (mm)	PR3 (mm)	RBD (%)	PW1 (mm)	PW3 (mm)
**BW208 wt**	44.3	854.4	2.00	4.12	11.5	142.7	75.7	45.7	39.7	16.7	4.3
**A1152**	44.8	841.6	1.85	**2.41** **	12.9	150.0	71.0	43.5	38.7	14.5	4.0
**Gazul wt**	48.8	832.8	1.77	2.78	15.0	170.8	85.3	60.0	29.8	17.7	6.0
**G613**	47.7	805.5	1.67	2.00	18.0***	162.5	81.7	**55.3** **	32.3	16.3	4.7
**G622**	49.5	817.1	2.34	2.78	16.2	141.7	**78.0** **	50.7***	**35.0** **	15.3***	4.3
**Podenco wt**	58.5	855.3	1.92	3.05	11.6	158.3	64.3	43.7	32.1	13.0	5.7
**G626**	**53.2** **	860.8	1.71	2.35	14.6***	141.7	75.0***	46.0	**38.7** **	15.0	4.0
**G845**	**52.1** **	854.8	1.80	2.14	15.9***	157.3	**70.0** **	46.7	33.2	15.7	5.0
**Arpain wt**	46.8	765.6	2.26	2.10	12.9	241.7	57.3	45.7	20.4	14.0	8.7
**G658**	47.2	**803.6** **	2.20	1.52	14.2	250.0	58.3	48.0	17.7	16.3	9.7
**G664**	47.9	794.3	3.08	1.72	**14.9** **	295.8***	59.7	49.7	**16.7** **	14.7	10.7
**Avr. Wild types**	49.6	827.0	1.99	3.01	12.8	178.4	70.7	48.8	30.5	15.3	6.2
**Avr. Transgenics**	48.9	825.4	2.09	**2.13** **	15.2***	185.6	70.5	48.6	30.3	15.4	6.0

The ratios of LMW/HMW (L/H) and Gliadins/Glutenins (Gli/Glu) were calculated from the RP-HPLC results described in Table 1. Means are significantly different (values in bold) to the respective wild-type line as determined by the LSD all-pairwise comparisons test as follow:

* P<0.05;

** P<0.01.

wt: wild type.

The mixing properties of the dough were determined by using the Mixograph, and the parameters MT (mixing time), PR1 (peak resistance), PR3 (height 3 min after the peak), RBD (resistance breakdown), PW1 (peak width) and PW3 (width 3 min after the peak) were calculated (Table 2 and Fig. S3). MT was not affected in most of the transgenic lines, and only the line G664 presented a significantly higher value of MT compared with the wild-type ‘Arpain’. The height of the curve PR1 was affected unequally in the transgenic lines of the different genotypes, being significantly lower in line G622 and significantly higher in lines G626 and G845, respect to their wild types. Similarly, PR3 was not affected in the same way in all the transgenic lines, and only the transgenic lines of the genotype ‘Gazul’ (G613 and G622) presented significantly lower values than the wild type. Consequently, the RBD, which is determined by the values PR1 and PR3, was affected in three transgenic lines (one of each genotype), two of which presented a significant increase respect to the wild type: G622 and G626, and one that presented a significant decrease: G664 (Table 2). The widths of the curve PW1 and PW3 were the most conserved parameters between wild types and transgenic lines, and only the PW1 in line G622 was significantly affected respect to the wild type ‘Gazul’.

However, for all the parameters measured using the Mixograph, none of them was significantly different in the average of transgenic lines respect to the average of wild types (Table 2), indicating that the mixing properties were not affected equally by the silencing of the γ-gliadins in the transgenic lines with different HMW-GS profile.

### Principal Components Analysis (PCA)

**Figure 3:**
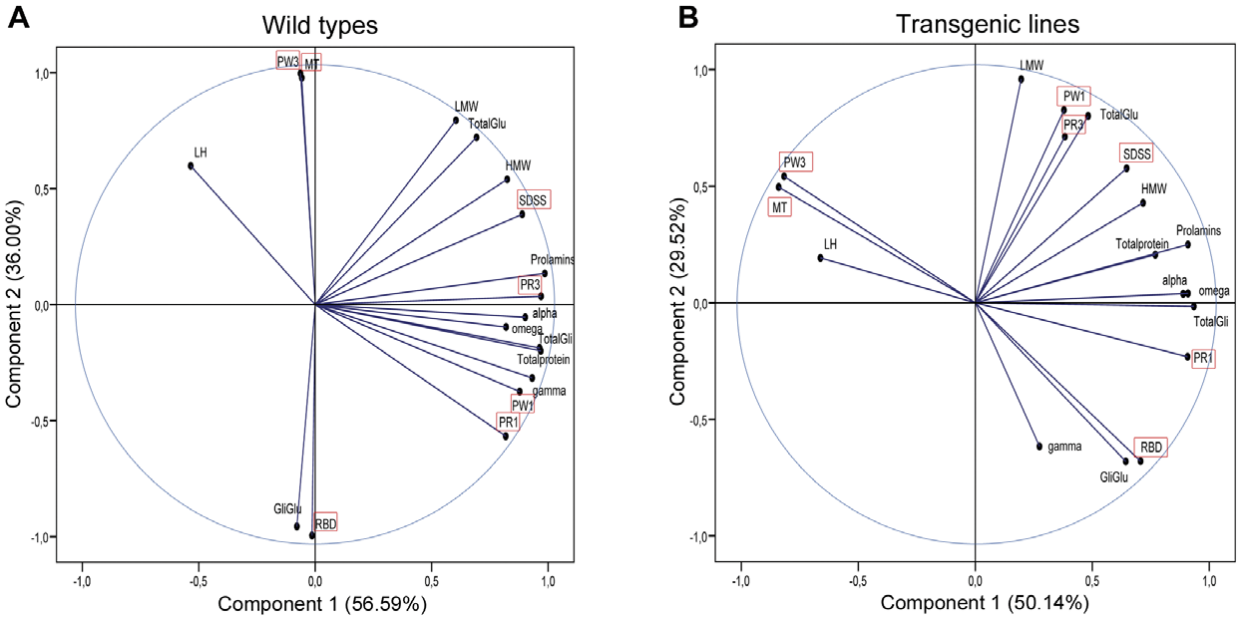
Principal component analysis (PCA) projections on axes 1 and 2. Wild types and the transgenic lines are represented in panel A and B, respectively. In each figure, the eigenvalues of the correlation matrix are symbolized as vectors representing traits that most influence each axis. Quality parameters (SDSS and Mixograph) are framed in a red square. Omega, ω-gliadin content; alpha, α-gliadin content; gamma, γ-gliadin content; TotalGli, total gliadins content; LMW, LMW-glutenin content; HMW, HMW-glutenin content; TotalGlu, total glutenin content; LH, LMW/HMW ratio; Prolamins, total prolamin content; GliGlu, Gliadins/Glutenins ratio; Totalprotein, total protein content.

Multivariate statistical analysis of the protein composition of the seeds and the quality parameters (Mixograph and SDSS analysis) of the transgenic and wild-type lines was carried out by principal components analysis (PCA) (Fig. 3) based on correlation matrix. Transgenic lines and wild types were studied separately in order to detect differences in their relationships between dough quality and grain composition in lines with presence and absence of the γ-gliadins fraction. Table S2 shows the significant (*p*<0.1) correlations found between the quality parameters and the protein composition of the grain for the wild types and the transgenic lines. In the PCA analysis of the wild types, the first two components explained 92.57% of the total variance (Fig. 3A). The first component, which represented 56.59% of the variability observed, was mainly correlated with total prolamins, total proteins and gliadin composition (α-, ω-, γ-gliadins and total gliadins), and also with the Mixograph parameters PR3, PR1 and PW1. The content of γ-gliadins was strongly correlated with PR1 and PR3 (Fig. 3A and Table S2). And the second component was mostly influenced positively by the MT and the PW3, and negatively by the Gli/Glu ratio and the RBD. Interestingly, the L/H ratio was not correlated with any of the studied characters in the wild-type lines, whereas total prolamins, total glutenins and HMW were positively correlated with the SDSS volume (Fig. 3A and Table S2). On the other hand, in the PCA of transgenic lines (Fig. 3B) the two first components explained 79.66% of the variance. The first component represented 50.14% of the total variance observed and, similarly to the PCA of wild types, was mainly correlated with total prolamins, α- and ω-gliadins, and total gliadins. However, in this case the amount of γ-gliadins was not influencing the first component and did not show significant correlations with any of the other parameters studied. LMW and total glutenin were the main factors influencing the second component and were positively correlated with PW1. For transgenic lines the L/H ratio was positively correlated with MT and PW3, and negatively correlated with RBD (Fig. 3B and Table S2).

## Discussion

Using RNAi technology the contents of α-gliadins [18], γ-gliadins [19] and all α-, γ- and ω-gliadins [20] were strongly down-regulated in bread wheat. In the present work, we report the introgression by conventional crossing of the down-regulation of γ-gliadins into three commercial bread wheat lines and the effects on the grain composition and mixing properties.

The dominant character of the RNAi-mediated silencing has been confirmed since the down-regulation of the γ-gliadins was observed in all the F1 progeny obtained from the different crossings. In addition, the inverted repeat sequence used to construct the transformation vectors, and which was cloned from *T. aestivum* cv. Bobwhite [19], was equally effective down-regulating the γ-gliadins in all the three commercial lines with different γ-gliadin profiles, producing significant reductions of this fraction in all the transgenic lines.

The down-regulation of γ-gliadins promoted increased synthesis of other prolamins, including the relative content of total glutenins. These results confirm those obtained by Pistón et al. [23] that reported that the compensatory mechanism may be governed by the availability of amino acids, and indicate that the mechanism of compensation is independent of the genotype.

During mixing, the gluten proteins are hydrated and homogeneously distributed throughout the dough, forming a three-dimensional gluten network structure due to the interactive behaviour of gliadins and glutenins through covalent and non-covalent bonding, which will determine the viscoelastic and gas holding properties of the dough. Therefore, dough mixing behaviour reflects the composition and structure of the gluten protein profile in the dough. A high Gli/Glu ratio will result in more extensible and relaxed doughs, whereas a high L/H ratio will decrease dough elasticity (reviewed by Hamer et al. [32]). For lines reported here, the L/H ratio was not significantly affected, whereas the Gli/Glu ratio was only reduced in the line A1152 and the average of transgenic lines respect to the wild types. These results agree with those for transgenic lines of cv. Bobwhite [23], for which the ratio Gli/Glu was unaffected in comparison to their wild type.

The three wild types used in the present study (‘Arpain’, ‘Gazul’ and ‘Podenco’) contain the pair 1D×5+1Dy10 of HMW glutenins and are considered high quality wheats, presenting medium/high W values in the Alveogram tests [33], [34]. The allelic pair of HMW subunits 1D×5+1Dy10 has been related to the production of large glutenin polymers and good bread-making quality (reviewed by Shewry et al. [1]).In the present work, gluten strength and baking quality were determined by the SDSS test [35] and the Mixograph. The sedimentation in the SDS solution theoretically results from the swelling of the glutenin strands [36], and high SDS sedimentation volumes have been associated with stronger gluten and superior bread-making quality [37], [38]. The SDSS test was significantly higher in four of the transgenic lines of the genotypes ‘Gazul’, ‘Podenco’ and ‘Arpain’, and also in the average of transgenic lines, but not in A1152. This different behaviour of the line A1152 may be explained by differences in the amount of total glutenins (and also HMW, total prolamins and total proteins), since they are the main factor influencing the SDSS volume as determined in the present study in the PCA and correlation analysis in both transgenic and wild-type lines. The SDSS volume has been already reported to be positively correlated with the content of HMW [39], total protein [35], and total glutenins [23].

In previous studies carried out in transgenic lines of two genotypes of the cv. Bobwhite (‘BW208’ and ‘BW2003’) the reduction of γ-gliadins had no major effect on the SDSS test [22], [23]. Results reported here confirm those results for cv. Bobwhite (BW208), but also show a different behaviour of the commercial lines, for which the increase of the SDSS value indicates increased gluten strength and quality of the transgenic lines respect to the wild types.

The Mixograph is a recording dough mixer, with the mixing bowl containing moving and fixed pins. In general, high dough strengths are characterised by long MT, high PR1 and high resistance to extension (high elasticity and low viscosity). A good balance between elasticity and viscosity is required for a good bread-making quality, and generally, high dough strengths are needed [8]. A good resistance to over-mixing is also required in the bread-making process. RBD and PW3 are, respectively, negatively and positively correlated with over-mixing tolerance.

The MT was found to be affected in only one line (G664) of the genotype ‘Arpain’, with most of the transgenic lines showing comparable values of MT than the wild types, and consequently similar dough strengths. The RBD was significantly increased in two transgenic lines, but decreased in the line G664, indicating that the reduction of γ-gliadins has not a clear effect on the mixing tolerance of the dough. In the PCA analysis in both the transgenic and wild-type lines the ratio Gli/Glu was found to be positively correlated with RBD, and negatively correlated with MT and PW3, indicating a weakening effect of the dough and a decrease in the over-mixing resistance with the increment of the proportion of gliadins. This observation fits with the results previously reported by Khan et al. [40], Fido et al. [8] and Uthayakumaran et al. [41] among others. In addition, an important change was observed in the correlations of the quality parameters MT, PW3 and RBD, that were more influenced by the L/H ratio and all the gliadin fractions (except the γ-gliadins) in the transgenic lines than in the wild-type lines. This indicates that in the transgenic lines with the γ-gliadins down-regulated the compensation observed in the rest of gliadins produced a weakening effect on the dough and a decrease of the resistance to over-mixing. However, the L/H ratio, which was not correlated with any quality parameter in the wild-type lines, appeared to positively influence the dough strength and the resistance to over-mixing in the transgenic lines. This agrees with the results obtained by Gupta and MacRitchie [42] and Sissons et al. [43], but not with those by Uthayakumaran et al. [44] that reported shorter MT with increasing ratios of LMW/HMW. The content of γ-gliadins in the wild-type lines was found to be positively correlated with some good quality parameters such as PR1 and PR3, but it did not influence any quality parameter in the transgenic lines. On the other hand the main factor influencing the SDSS volume in both transgenic and wild-type lines was the total glutenin content, with also HMW, total prolamins and total proteins being important. This indicates that glutenins, and more particularly HMW glutenins, are main factors influencing the baking and mixing properties of the dough, and play an important role in the formation of the gluten network. The mixing properties were previously determined by the Mixograph in 18 transgenic lines of cv. Bobwhite with reduced contents of γ-gliadins [23], concluding that the reduction of the γ-gliadin content does not have a major effect on the Mixograph parameters. However, in the present study, although none of the mixing parameters were significantly affected in the average of transgenic lines, most of the changes observed in the mixing properties of the transgenic lines occurred in the genotypes ‘Gazul’, ‘Podenco’ and ‘Arpain’ and not in the transgenic line of the cv. Bobwhite (A1152). Finally, in some parameters such as PR1 and RBD the reduction of γ-gliadins had opposite effects depending on the transgenic line, indicating the influence of the genotype (and specifically the HMW-GS composition) on the changes observed in the quality as consequence of the silencing of γ-gliadins.

In the present work, γ-gliadins have been down-regulated in three commercial lines by RNAi-mediated gene silencing. Gliadins have been reported to decrease the overall dough strength and stability when added to base flours [45]–[48] and our results confirm this since the reduction of the γ-gliadins affected positively to the dough strength (SDSS volume) and other mixing parameters in some of the lines. However, gliadins may also play an important role in the formation of the three dimensional viscoelastic structures. Fido et al. [8] reported that gliadins may be involved in the formation of extensive gluten film networks through covalent and non-covalent bonding with other gluten proteins. In the α- and γ-gliadins three and four intramolecular disulphide bonds are formed, respectively [49], [50], whereas in the ω-gliadins no cysteine or methionine are present [51], [52] and therefore no disulphide bonds exist. Kasarda [53] suggested that gliadins having an odd number of cysteine residues are able to retain one free cysteine after the formation of intra-molecular disulphide bonds, which could contribute to intermolecular disulphide bonding. Considering this, if the γ-gliadins have any role in the formation of the three dimensional gluten structure, this might be compensated by the increase of α-gliadins, and consequently, increases in the composition of α-gliadins in the transgenic lines, together with increased amounts of HMW and LMW glutenins, might explain the different behaviour observed in the transgenic lines described in the present study.

### Conclusions

The compensatory mechanism promoted by the down-regulation of γ-gliadins is highly stable and independent of the genotype. As consequence, the glutenin content is increased and the ratio Gli/Glu reduced in the transgenic lines, provoking an increase of the dough strength and a decrease of the extensibility. The correlations between grain composition and quality parameters were studied separately in the wild types and transgenic lines by the PCA analysis, showing differences in the parameters that affect the dough quality in both types.

In addition, the increase of the SDSS value in the transgenic lines of ‘Gazul’, ‘Podenco’ and ‘Arpain’ indicates increased quality respect to the wild types. Although the reduction of γ-gliadins seems not to have a direct effect on the mixing and bread-making properties, the compensatory effect on the synthesis of the other prolamins has resulted in stronger doughs with improved over-mixing resistance in some of the transgenic lines reported in the present work.

## Supporting Information

Figure S1 **RP-HPLC chromatograms of gliadin and glutenin extracts from wild types and transgenic lines.**
(TIF)

Figure S2 **Mature seeds from wild types and transgenic lines.**
(TIF)

Figure S3 **Mixograph curves of the doughs prepared from flours of the non-transformed controls and the transgenic lines.**
(TIF)

Table S1 **Protein and starch composition of transgenic lines (Mean ± standard error).**
(DOCX)

Table S2 **Pearson’s correlation coefficients between protein composition and quality parameters.**
(DOCX)
