# Supplementary figures and images for: The Introgression of RNAi Silencing of γ-Gliadins into Commercial Lines of Bread Wheat Changes the Mixing and Technological Properties of the Dough
Source: PLoS One. 2012 Sep 24;7(9):e45937. doi: 10.1371/journal.pone.0045937 (PMC3454332; doi:10.1371/journal.pone.0045937)

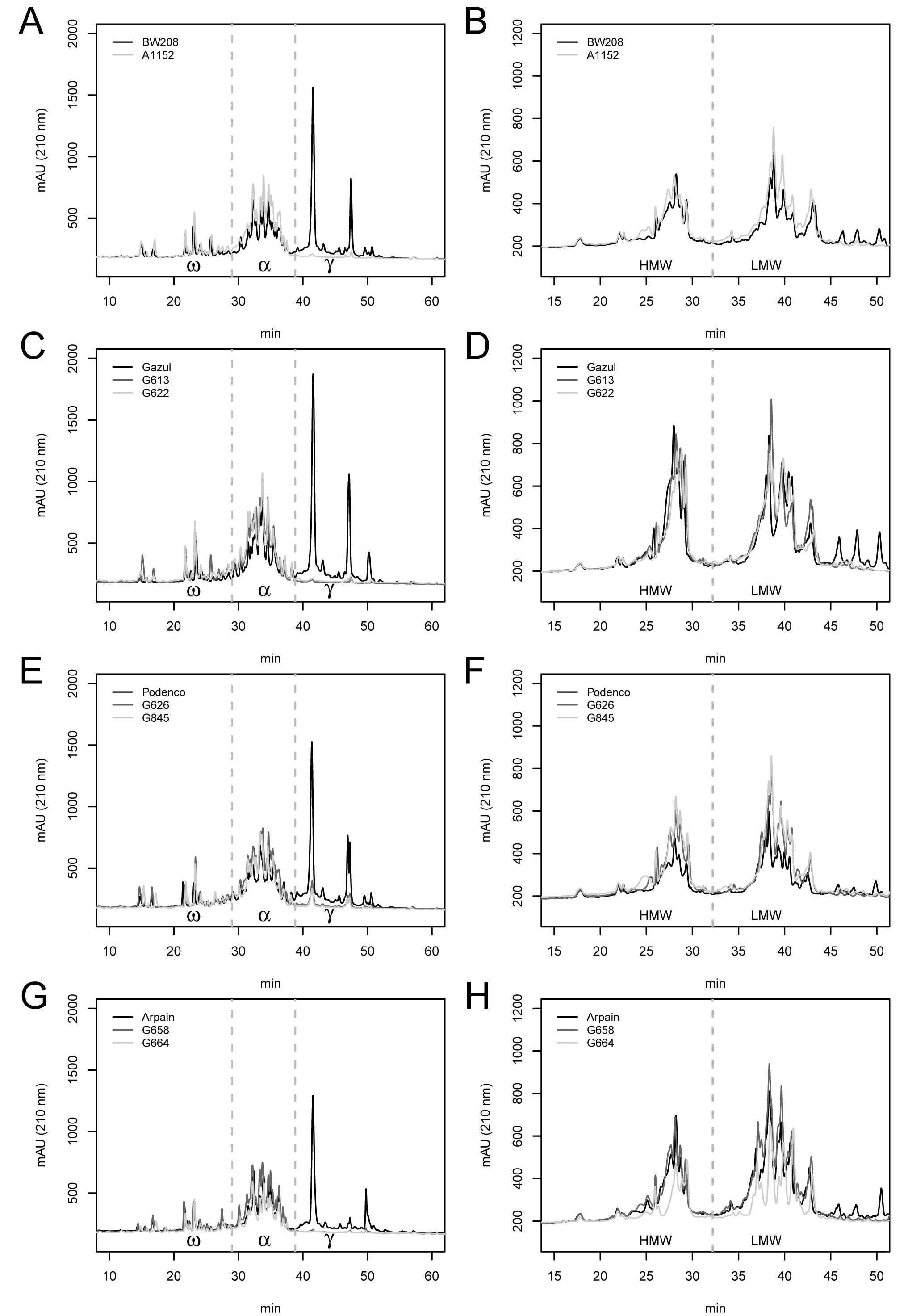

Supplement: Figure S1 — RP-HPLC chromatograms of gliadin and glutenin extracts from wild types and transgenic lines. (TIF) [file pone.0045937.s001.tif]

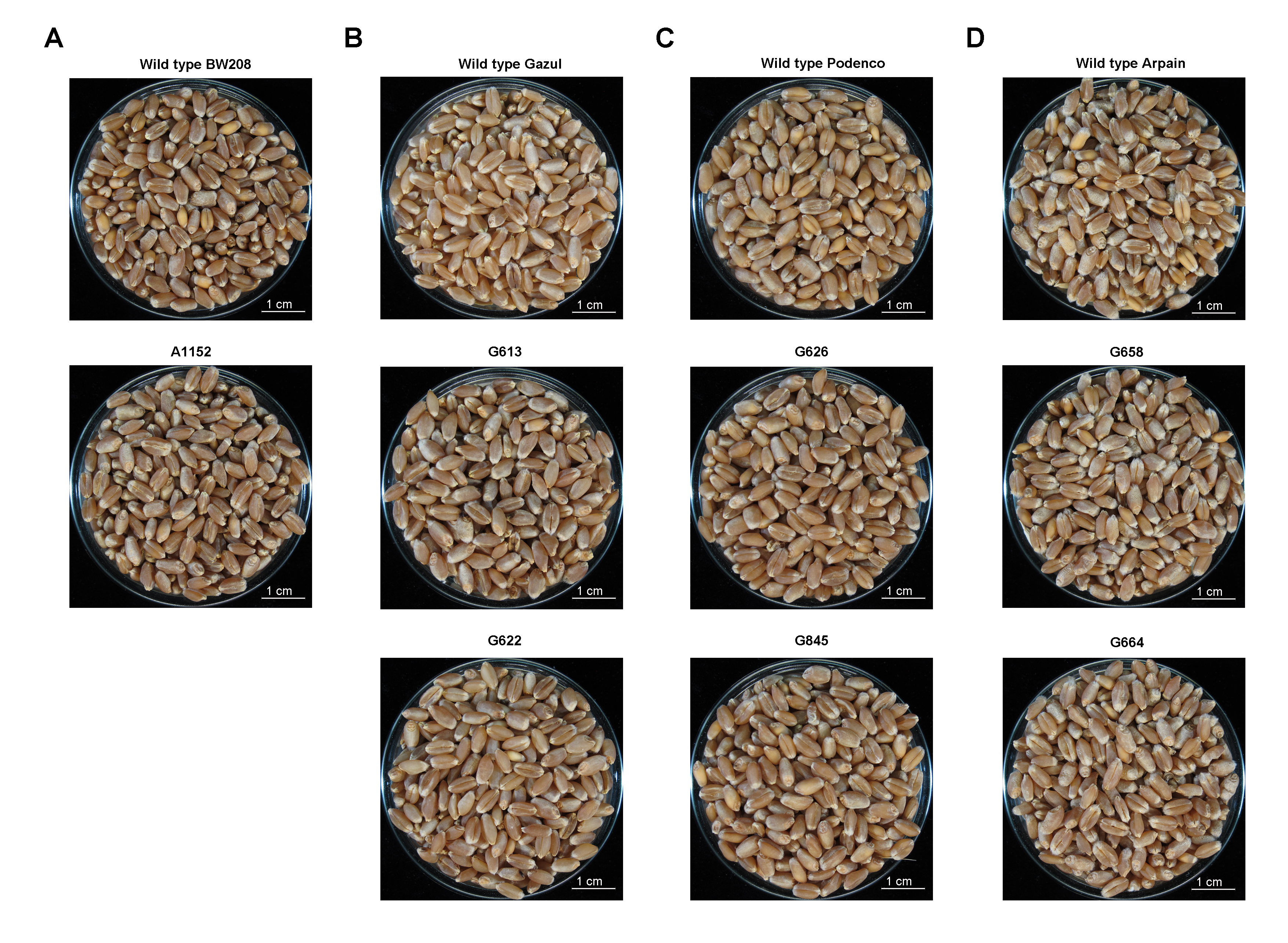

Supplement: Figure S2 — Mature seeds from wild types and transgenic lines. (TIF) [file pone.0045937.s002.tif]

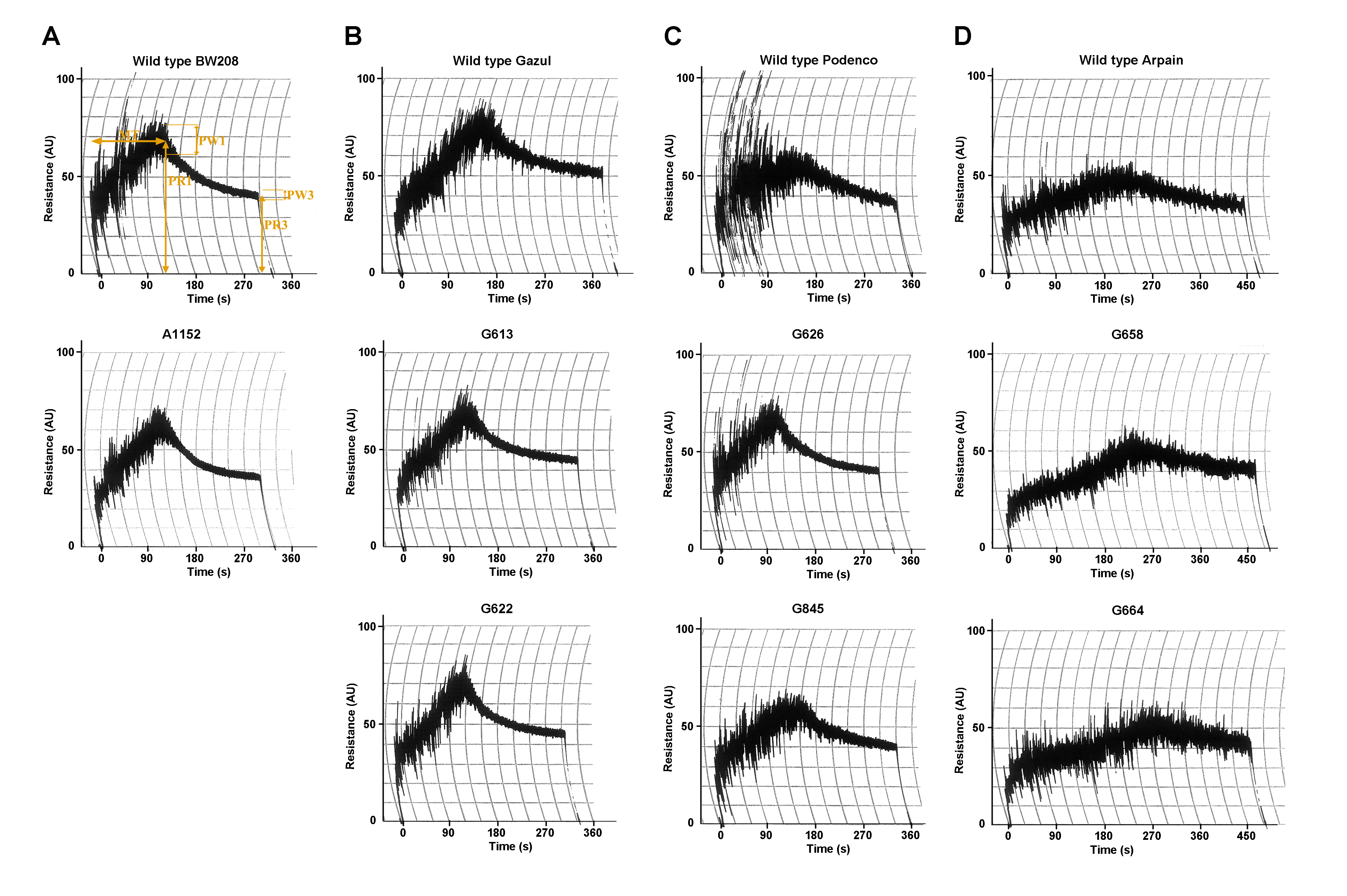

Supplement: Figure S3 — Mixograph curves of the doughs prepared from flours of the non-transformed controls and the transgenic lines. (TIF) [file pone.0045937.s003.tif]
