# Supplementary material for: The Introgression of RNAi Silencing of γ-Gliadins into Commercial Lines of Bread Wheat Changes the Mixing and Technological Properties of the Dough
Source: PLoS One. 2012 Sep 24;7(9):e45937. doi: 10.1371/journal.pone.0045937 (PMC3454332; doi:10.1371/journal.pone.0045937)
Supplement: Table S1 — Protein and starch composition of transgenic lines (Mean ± standard error). (DOCX) [file pone.0045937.s004.docx]

**Table S1.** Protein and starch composition of transgenic lines (Mean ± standard error).

| **Line** | **Gliadin ± SE** (μg mg^-1^ flour) | | | | | | | | | | | |  | **Glutenin ± SE** (μg mg^-1^ flour) | | | | | | | | | **Prolamins ± SE** (μg mg^-1^ flour) | | | **Total protein ± SE** (%) | | | **Total starch ± SE** (%) | | |
| --- | --- | --- | --- | --- | --- | --- | --- | --- | --- | --- | --- | --- | --- | --- | --- | --- | --- | --- | --- | --- | --- | --- | --- | --- | --- | --- | --- | --- | --- | --- | --- |
|  | **ω** | | | **α** | | | **γ** | | | **Total** | | |  | **LMW** | | | **HMW** | | | **Total** | | |  |  |  |  |  |  |  |  |  |
| A1152 | 9.90 | ± | 1.37 | 36.86 | ± | 7.00 | 1.06 | ± | 0.25 | 47.82 | ± | 8.62 |  | 12.87 | ± | 2.17 | 6.90 | ± | 0.90 | 19.77 | ± | 3.07 | 67.59 | ± | 11.59 | 12.03 | ± | 0.45 | 53.68 | ± | 0.97 |
| G613 | 11.21 | ± | 1.31 | 42.18 | ± | 7.74 | 1.01 | ± | 0.44 | 54.40 | ± | 9.48 |  | 17.57 | ± | 4.44 | 10.29 | ± | 2.03 | 27.86 | ± | 6.45 | 82.26 | ± | 15.92 | 13.88 | ± | 0.99 | 53.76 | ± | 1.80 |
| G622 | 12.96 | ± | 4.48 | 48.04 | ± | 13.26 | 1.29 | ± | 0.25 | 62.29 | ± | 17.99 |  | 15.30 | ± | 1.15 | 7.30 | ± | 1.34 | 22.60 | ± | 0.77 | 84.89 | ± | 17.79 | 14.07 | ± | 0.43 | 57.14 | ± | 1.70 |
| G626 | 7.69 | ± | 0.06 | 30.6 | ± | 3.16 | 3.04 | ± | 0.82 | 41.32 | ± | 3.92 |  | 13.32 | ± | 2.20 | 7.77 | ± | 1.23 | 21.09 | ± | 3.42 | 59.01 | ± | 3.21 | 12.69 | ± | 0.17 | 54.19 | ± | 0.16 |
| G845 | 9.97 | ± | 1.49 | 43.03 | ± | 7.74 | 0.52 | ± | 0.05 | 53.52 | ± | 9.26 |  | 15.88 | ± | 2.01 | 8.89 | ± | 1.39 | 24.77 | ± | 3.32 | 78.29 | ± | 12.57 | 12.67 | ± | 0.60 | 54.20 | ± | 1.67 |
| G658 | 8.18 | ± | 0.98 | 30.85 | ± | 4.47 | 0.47 | ± | 0.10 | 39.50 | ± | 5.54 |  | 17.86 | ± | 2.56 | 8.18 | ± | 1.41 | 26.04 | ± | 3.96 | 65.54 | ± | 9.50 | 11.98 | ± | 0.39 | 54.87 | ± | 1.04 |
| G664 | 6.63 | ± | 0.75 | 25.89 | ± | 5.84 | 0.32 | ± | 0.08 | 32.84 | ± | 6.66 |  | 14.65 | ± | 3.66 | 5.20 | ± | 1.59 | 19.85 | ± | 4.53 | 52.69 | ± | 10.77 | 12.48 | ± | 0.33 | 53.94 | ± | 0.86 |
| Avr. Transgenics | 9.51 | ± | 0.81 | 36.78 | ± | 3.05 | 1.10 | ± | 0.20 | 47.38 | ± | 3.89 |  | 15.35 | ± | 0.97 | 7.79 | ± | 0.56 | 23.14 | ± | 1.42 | 70.04 | ± | 4.86 | 12.83 | ± | 0.24 | 54.54 | ± | 0.48 |
