# Supplementary material for: The Introgression of RNAi Silencing of γ-Gliadins into Commercial Lines of Bread Wheat Changes the Mixing and Technological Properties of the Dough
Source: PLoS One. 2012 Sep 24;7(9):e45937. doi: 10.1371/journal.pone.0045937 (PMC3454332; doi:10.1371/journal.pone.0045937)
Supplement: Table S2 — Pearson’s correlation coefficients between protein composition and quality parameters. (DOCX) [file pone.0045937.s005.docx]

**Table S2.** Pearson’s **c**orrelation coefficients between protein composition and quality parameters.

|  | **SDSS** | |  | **MT** | |  | **PR1** | |  | **PR3** | |  | **RBD** | |  | **RBD** | |  | **PW1** | |  | **PW3** | |
| --- | --- | --- | --- | --- | --- | --- | --- | --- | --- | --- | --- | --- | --- | --- | --- | --- | --- | --- | --- | --- | --- | --- | --- |
|  | **wt** | **t** |  | **wt** | **t** |  | **wt** | **t** |  | **wt** | **t** |  | **wt** | **t** |  | **wt** | **t** |  | **wt** | **t** |  | **wt** | **t** |
| **ω-gliadins** | *NS* | *NS* |  | *NS* | **-0.67*** |  | *NS* | **0.73*** |  | *NS* | *NS* |  | NS | NS |  | *NS* | *NS* |  | **0.92*** | *NS* |  | *NS* | *NS* |
| **α-gliadins** | *NS* | *NS* |  | *NS* | **-0.71*** |  | *NS* | *NS* |  | *NS* | *NS* |  | NS | NS |  | *NS* | *NS* |  | **0.94*** | *NS* |  | *NS* | **-0.67*** |
| **γ-gliadins** | *NS* | *NS* |  | *NS* | *NS* |  | **0.96**** | *NS* |  | **0.94*** | *NS* |  | NS | NS |  | *NS* | *NS* |  | *NS* | *NS* |  | *NS* | *NS* |
| **Total Gliadins** | *NS* | *NS* |  | *NS* | **-0.76**** |  | **0.89*** | **0.77**** |  | **0.90*** | *NS* |  | NS | NS |  | *NS* | *NS* |  | **0.97**** | *NS* |  | *NS* | **-0.72*** |
| **LMW** | *NS* | *NS* |  | *NS* | *NS* |  | *NS* | *NS* |  | *NS* | *NS* |  | NS | NS |  | *NS* | *NS* |  | *NS* | **0.94***** |  | *NS* | *NS* |
| **HMW** | **0.98**** | *NS* |  | *NS* | *NS* |  | *NS* | *NS* |  | *NS* | *NS* |  | NS | NS |  | *NS* | *NS* |  | *NS* | **0.80**** |  | *NS* | *NS* |
| **Total glutenins** | **0.90*** | **0.68*** |  | *NS* | *NS* |  | *NS* | *NS* |  | *NS* | *NS* |  | NS | NS |  | *NS* | *NS* |  | *NS* | **0.98***** |  | *NS* | *NS* |
| **L/H** | *NS* | *NS* |  | *NS* | **0.80**** |  | *NS* | *NS* |  | *NS* | *NS* |  | NS | **-0.73*** |  | *NS* | **0.73*** |  | *NS* | *NS* |  | *NS* | **0.78**** |
| **Total prolamins** | **0.90*** | *NS* |  | *NS* | *NS* |  | *NS* | **0.68*** |  | **0.94*** | *NS* |  | NS | NS |  | *NS* | *NS* |  | *NS* | *NS* |  | *NS* | *NS* |
| **Gli/Glu** | *NS* | *NS* |  | *NS* | **-0.82**** |  | *NS* | **0.72*** |  | *NS* | *NS* |  | **0.98**** | **0.85**** |  | **0.98**** | **0.85**** |  | *NS* | *NS* |  | **-0.94*** | **-0.83**** |
| **Total protein** | *NS* | **0.85**** |  | *NS* | *NS* |  | **0.91*** | **0.78**** |  | **0.97**** | **0.76**** |  | NS | NS |  | NS | NS |  | *NS* | *NS* |  | *NS* | *NS* |

*, *p*<0.1; **, *p*<0.01; ***, *p*<0.001. wt: wild types; t: transgenic lines
